# Early Fetal Growth Restriction of Both Twins in a Transgender Man

**DOI:** 10.1155/2022/2905539

**Published:** 2022-09-08

**Authors:** Alicia Martínez-Varea, Clara Martínez-Sáez, María Patrocinio Tarrazó-Millet, Vicente Diago-Almela

**Affiliations:** ^1^Department of Obstetrics and Gynaecology, La Fe University and Polytechnic Hospital, Avenida Fernando Abril Martorell 106, 46026 Valencia, Spain; ^2^Department of Obstetrics and Gynaecology, Dr. Peset University Hospital, Avenida de Gaspar Aguilar 90, 46017 Valencia, Spain

## Abstract

Gender dysphoria affects 0.5% of people worldwide. Transgender men display a divergence between the female genetic sex and the gender male identity. To the best of our knowledge, we describe the first case report with regard to a transgender man with a dichorionic diamniotic twin pregnancy obtained by artificial insemination using donor sperm as a monoparental family, presenting early fetal growth restriction of both twins. The patient is a 35-year-old transgender man who had previously received gender-affirming hormone therapy based on testosterone for five years and had a prior bilateral mastectomy as gender-affirming surgery. Whether assisted reproductive techniques have any influence on obstetrical outcomes among these patients and whether prior long-term intake of gender-affirming hormone therapy has an impact on pregnancy and obstetrical outcomes remain to be elucidated.

## 1. Introduction

Gender dysphoria, a disparity between genetically determined sex and gender identity, affects roughly 0.5% of people worldwide [1]. Transgender men display a divergence between the female genetic sex and the gender male identity [1]. The gender-affirming process often includes gender-affirming hormone therapy (GAHT) [1, 2] as well as gender-affirming surgery (GAS) [1, 3]. GAHT is based on the use of testosterone and its derivatives to provide transgender men the longing masculine appearance [1, 2]. GAS may include mastectomy, genital reconstruction, and hysterectomy with or without salpingo-oophorectomy [1, 3, 4]. Interestingly, it has been described that the vast majority of transgender men retain their female reproductive organs and, hence, their potential ability to become pregnant [4, 5].

The precise prevalence of pregnancy among transgender men is unknown [5]. Nonetheless, expert opinion suggests that the number of pregnant transgender men is increasing [4, 5]. The impact on fertility and pregnancy outcomes of long-term testosterone treatment among transgender men is unknown. Additionally, data regarding pregnancy in transgender pregnant men are still limited. To the best of our knowledge, we describe the first case report with regard to a transgender man with a dichorionic diamniotic twin pregnancy obtained by artificial insemination presenting early fetal growth restriction of both twins.

## 2. Case Report

The patient is a 35-year-old transgender man with a dichorionic diamniotic twin pregnancy obtained by the second artificial insemination using donor sperm as a monoparental family. Previous medical background includes a childhood history of asthma without the requirement of current treatment. He had previously received GAHT based on testosterone (intramuscular testosterone cypionate 250 mg/mL every 18 days) for 5 years. This therapy was ceased 3 months prior to initiating the fertility treatment. Prior surgical history includes gastric bypass surgery, abdominoplasty, and bilateral mastectomy as GAS.

The patient weight at the beginning of pregnancy was 70 kilograms, and the height was 164 centimeters, resulting in a body mass index mass of 26. He smoked 20 cigarettes per day, which was reduced to 2-3 cigarettes daily after becoming pregnant. During pregnancy, the transgender man received daily pregnancy vitamins which included 400 micrograms of folic acid and 150 milligrams of acetylsalicylic acid to prevent early preeclampsia.

The dichorionic diamniotic twin pregnancy was confirmed by ultrasound at 10 weeks gestation. The risk for trisomy 21, 13, and 18 was low at the combined screening performed at 12 weeks gestation. The morphological fetal ultrasound performed at 20 weeks gestation was within normal limits. The pregnancy was uneventful until 29^+0^ weeks gestation, when selective fetal growth restriction of the first twin was diagnosed. Thus, the patient underwent weekly monitoring. At 31^+5^ weeks gestation, early fetal growth restriction of both twins was detected. Ultrasound study revealed biometry of biparietal diameter (BPD) 75.3, head circumference (HC) 266.0, abdominal circumference (AC) 206.0, femur length (FL) 55.2, with an estimated fetal weight of 1046 grams (<percentile 1 for gestational age, Hadlock) of the first twin, which was in cephalic presentation and presented normal amniotic fluid (maximum vertical pocket of 3.0 cm). The second twin was also in cephalic presentation and displayed biometry of BPD 69.5, HC 283.7, AC 242.4, and FL 55.3, with an estimated fetal weight of 1300 grams (percentile 1 for gestational age, Hadlock), as well as normal amniotic fluid (maximum vertical pocket of 4.8 cm). The color Doppler study showed an intermittent absent end-diastolic flow of the umbilical artery of both twins. The pulsatility index of the middle cerebral artery of both twins was within normal limits (1.62 and 2.13, respectively). The blood flow of the ductus venosus was normal in both twins, as well as the mean pulsatility index of the uterine arteries (0.46). Cardiotocography disclosed a stable baseline of approximately 130 beats per minute and a reassuring variability of both twins. Due to the early fetal growth restriction with the intermittent absent end-diastolic flow of the umbilical artery of both twins, the patient was admitted into the hospital in order to initiate fetal lung maturation with betamethasone 2 mL/24 h, 2 doses, and to monitor daily both fetuses with cardiotocography and ultrasound. During the hospital admission, the patient showed a blood pressure within normal limits, being the blood and urine tests nonremarkable. The soluble fms-like tyrosine kinase-1/placental growth factor (sFlt-1/PlGF) ratio was 12 at 31^+5^ weeks gestation.

At 32^+2^ weeks gestation the fetal color Doppler study revealed a persistent absent end-diastolic flow of the umbilical artery in both fetuses with growth restriction. Given that the American College of Obstetricians and Gynecologists suggest delivery between 32^+0^ and 37^+6^ weeks gestation in growth-restricted fetuses with additional risk factors for adverse outcomes, such as abnormal umbilical artery Doppler [6], the risk of emergent cesarean section at labor exceeds 50% in fetuses with growth restriction and absent end-diastolic flow of the umbilical artery [7], and the unknown effects of long-term GAHT on myometrium and placenta, an elective cesarean delivery was performed as individualized management. The patient agreed and signed the informed consent. The first newborn was a male of 1250 grams, who displayed an Apgar score of 10/10/10, an arterial pH of 7.39, and a venous pH of 7.39. The second newborn was a female of 1390 grams, who showed an Apgar score of 10/10/10, an arterial pH of 7.30, and a venous pH of 7.36. The transgender man underwent an uneventful postpartum recovery and was discharged 48 hours after the cesarean section. Both newborns were discharged four weeks after birth and have not presented major morbidity.

## 3. Discussion

Transgender men have previously received scarce attention with respect to reproductive health and obstetrical care. Although the number of transgender individuals is rising [8], there is a lack of information regarding fertility, pregnancy, and neonatal outcomes among transgender people. Additionally, the impact of GAHT on fertility and pregnancy outcomes is unknown. This case report presents a dichorionic diamniotic twin pregnancy obtained by artificial insemination in a transgender man with early fetal growth restriction and absent end-diastolic flow of the umbilical artery of both twins. We hypothesize that the history of long-term use of testosterone as GAHT may have a potential impact on myometrium and on the subsequent placental development and function, as well as on fetal growth.

It has been described that GAHT in transgender men entails breast atrophy without an associated increased risk of breast cancer [2]. Moreover, the androgen treatment in transgender men induces ovarian effects after 6 months of therapy that consists of modifications of ovarian morphology that simulate the ovarian appearance seen in women with polycystic ovary syndrome but without a variation on antral follicle count [2]. Long-term androgen therapy in transgender men induces a similar metabolic profile to cisgender men with regard to lipid profile, insulin resistance, and mortality [2]. Although data regarding cardiometabolic risk is reassuring, some authors have reported conflicting results [2]. Actually, given that GAHT increases leukocyte-endothelium interactions and proinflammatory cytokines, it has been suggested that cardiovascular risk should be monitored in transgender men [9]. Concerning pregnancy in transgender men, a cross-sectional study based on a web-based survey has reported that pregnancy, delivery, and neonatal outcomes do not differ according to prior testosterone use as GAHT. A total of 36 out of 41 patients (88%) used their own oocytes, the mean gestational age at delivery was 38+6 weeks gestation, and 29 out of 41 patients (71%) underwent vaginal delivery [10]. Although patients with previous use of GAHT underwent more cesarean deliveries compared to those without the prior intake of GAHT (36% [9/25] versus 19% [3/16], respectively), the difference was not statistically significant [10]. A subsequent cross-sectional study based on a quantitative survey is in line with the finding that only a minority (23%) of transgender individuals deliver by cesarean section, although prior GAHT is not taken into account [11]. Both studies lack the comparison of pregnancy outcomes between singleton and multiple pregnancies, as well as between spontaneously conceived gestations and those obtained through assisted reproductive techniques. Ultimately, the impact of prior GAHT on labor and obstetrical outcomes is largely unknown [5, 11].

Regarding psychological aspects of pregnancy in transgender men, it is noteworthy that the interruption of GAHT before pregnancy accompanied by the increase of female hormones due to pregnancy status may increase gender dysphoria [4, 5, 12]. Moreover, it is crucial to closely monitor transgender men during postpartum in order to rule out postpartum depression, given that baseline depression among transgender individuals is higher compared to adult average and a number of these patients experience a lack of social support as well as individual loneliness during pregnancy and parenting [4, 12, 13].

Transgender men have to decide whether to reinitiate testosterone during the postpartum period [4]. For those who have not undergone a mastectomy as GAS and desire to breastfeed, it has been shown that 100 mg subcutaneous testosterone pellet is not associated with a marked increase of milk testosterone levels [14]. Although subcutaneous testosterone cypionate increases milk testosterone levels, testosterone displays low oral bioavailability due to its extensive first-pass metabolism, and apparently, it does not increase serum testosterone levels in breastfed infants [14]. Breastfed infants appear not to be adversely affected by transgender paternal GAHT [14]. High doses of testosterone are able to suppress lactation [4, 14]. On the other hand, for transgender men who do not seek to breastfeed, data to guide the new beginning of GAHT after delivery is limited [5]. It has been suggested to wait between 4 and 6 weeks after delivery [5].

It is essential to address postpartum contraception with transgender men [5, 13], given that ovulation and pregnancy may still occur while amenorrheic with GAHT [15] and testosterone induces virilization during embryogenesis [5]. All contraceptive options may be offered to transgender men because testosterone is not a contraindication to any form of contraception [15].

In conclusion, the number of transgender individuals is increasing [8], and data regarding fertility, pregnancy, and neonatal outcomes among transgender people is limited. Notably, the impact of GAHT on labor and obstetrical outcomes is largely unknown [5, 11]. Thus, the need for obstetrician-gynecologists and reproductive specialists to improve care for transgender men should be highlighted. Further prospective studies are needed in order to clarify fertility and pregnancy outcomes among transgender men and the impact, if any, of prior GAHT. It has been presented a dichorionic diamniotic twin pregnancy in a transgender man obtained by artificial insemination with early fetal growth restriction and absent end-diastolic flow of the umbilical artery of both twins. The patient underwent GAHT for 5 years before pregnancy. Whether assisted reproductive techniques have any influence on obstetrical outcomes among these patients and whether prior long-term intake of GAHT has an impact on fertility, pregnancy, and obstetrical outcomes remain to be elucidated.

## Data Availability

Data regarding this case report are in the clinical history of the patient.
